# Pyrrolizidine Alkaloids in Foods, Herbal Drugs, and Food Supplements: Chemistry, Metabolism, Toxicological Significance, Analytical Methods, Occurrence, and Challenges for Future

**DOI:** 10.3390/toxins16020079

**Published:** 2024-02-02

**Authors:** Bruna Tábuas, Sílvia Cruz Barros, Catarina Diogo, Carlos Cavaleiro, Ana Sanches Silva

**Affiliations:** 1 Faculty of Pharmacy, University of Coimbra, Polo III, Azinhaga de Santa Comba, 3000-548 Coimbra, Portugalcavaleir@ff.uc.pt (C.C.); 2 National Institute for Agricultural and Veterinary Research (INIAV), I.P, 4485-655 Vila do Conde, Portugal; 3 Chemical Process Engineering and Forest Products Research Centre (CIEPQPF), University of Coimbra, Rua Sílvio Lima, 3030-790 Coimbra, Portugal; 4 Center for Study in Animal Science (CECA), Institute of Sciences, Technologies and Agro-Environment of the University of Porto (ICETA), University of Porto, 4501-401 Porto, Portugal; 5 Associate Laboratory for Animal and Veterinary Sciences (AL4AnimalS), 1300-477 Lisbon, Portugal

**Keywords:** pyrrolizidine alkaloids, toxicology, metabolism, chromatography, mass spectrometry, extraction, food supplements, dried plants

## Abstract

Consumers are increasingly seeking natural alternatives to chemical compounds, including the use of dried aromatic plants as seasonings instead of salt. However, the presence of pyrrolizidine alkaloids (PAs) in food supplements and dried plants has become a concern because of their link to liver diseases and their classification as carcinogenic by the International Agency for Research on Cancer (IARC). Despite European Union (EU) Regulation (EU) 2023/915, non-compliance issues persist, as indicated by alerts on the Rapid Alert System for Food and Feed (RASFF) portal. Analyzing PAs poses a challenge because of their diverse chemical structures and low concentrations in these products, necessitating highly sensitive analytical methods. Despite these challenges, ongoing advancements in analytical techniques coupled with effective sampling and extraction strategies offer the potential to enhance safety measures. These developments aim to minimize consumer exposure to PAs and safeguard their health while addressing the growing demand for natural alternatives in the marketplace.

## 1. Introduction

Pyrrolizidine alkaloids (PAs) are a group of natural compounds that are present in several plant families, among which the most predominant are Boraginaceae, Asteraceae, and Fabaceae, and are produced as a defense response against herbivores [1]. PAs are known for their toxicity and for posing an elevated risk to human and animal health when consumed in large quantities over long periods of time. Several studies have reported their water and chronic toxicity, hepatotoxicity, genotoxicity, and carcinogenicity in both animals and humans [2]. Consumption of PAs due to their liver toxicity has been indicated as a major cause of hepatic veno-occlusive disease (HVOD), which can lead to cirrhosis or, eventually, total liver failure [3]. Excessive consumption of PAs is also associated with pulmonary hypertension, cardiac hypertrophy, kidney damage, and even death [3,4].

Chemically, PAs are composed of a pyrrolizidine ring, which consists of a cyclic pyrrolizidine core fused to a necine base. The necine base can vary, giving rise to different types of PAs with different levels of toxicity. There are now over 600 known types of PAs identified in over 6000 different plant species [5].

PAs can enter the human food chain through various routes, including contamination of crops during cultivation, harvesting, processing, and storage. They are present in food products such as cereals, honey, teas, herbal products, spices, and food supplements. Contamination can occur through direct or indirect contact [6,7].

The European Commission (EC) has set the limit for the maximum levels of contaminants allowed in food products to ensure consumer safety regarding different groups of compounds, namely PAs. Researchers continue to study PAs to better understand their occurrence, toxicology, and potential health risks. Methodologies have been developed over the years to improve the detection and quantification of these compounds, and authorities have focused on exploring mitigation strategies and promoting awareness among consumers, food producers, and regulatory bodies [2,8].

The consumption of PAs and their N-oxides (Pyrrolizidine Alkaloids N-Oxide, PANOS) is an urgent and major public health concern that needs to be addressed; therefore, analytical methods that can identify and quantify very low concentrations of these compounds need to be developed [4]. The present monograph includes a review of the techniques used in the extraction, detection, and quantification of PAs and their N-oxides in the last few years (2017–2023) in food supplements and dried plants. Moreover, their analytical challenges and future perspectives are addressed.

## 2. Pyrrolizidine Alkaloids

### 2.1. Data Sources and Search Strategy

The literature search was conducted online, using the following databases: SCOPUS and Science Direct. The literature search included academic articles published in English mainly published in the last decade. The keywords search was conducted using the words “Pyrrolizidine alkaloids” in association with one of the following words: toxicology; metabolism; chromatography; mass spectrometry; extraction; food supplements; dried plants; foods; and herbal drugs.

### 2.2. Chemistry of PAs

**Figure 1:**
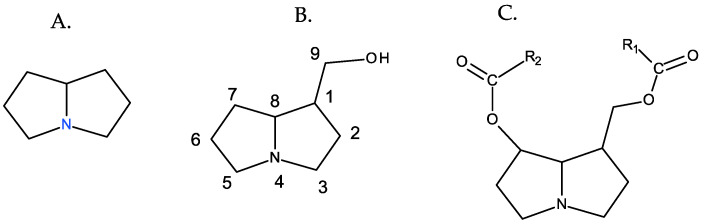
Fundamental structures of PAs. From left to right: (**A**) pyrrolizidine, (**B**) 1-hydroxymethylpyrrolizidine (necine), and (**C**) necine diester.

PAs are organic compounds characterized by the heterocyclic nucleus of pyrrolizidine, hydroxymethylated at position 1 (necine), and generally esterified with aliphatic acids, which are generically referred as necic acids [9] (Figure 1).

**Figure 2:**
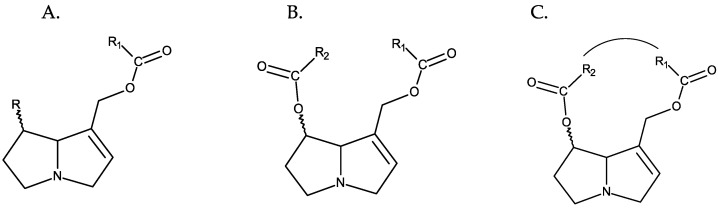
Different types of esterification of PAs. From left to right: (**A**) monoester, (**B**) open-chain diester, and (**C**) cyclic diester.

In most cases, the necine base, in addition to the hydroxymethyl at C-1, is also hydroxylated at C-7. This hydroxyl can also be esterified, and depending on whether esterification occurs at one or both hydroxyl groups (positions C-7 and C-9), PAs can be monoesters or diesters and, in the latter case, open-chain esters or cyclic esters [9] (Figure 2).

**Figure 3:**
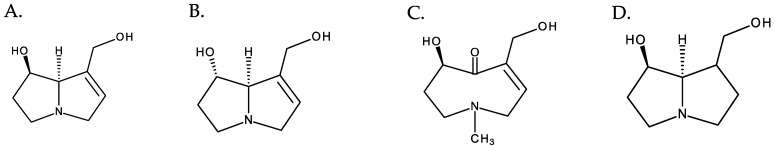
Structures of the most common necine bases. From left to right: (**A**) retronecine, (**B**) heliotridine, (**C**) otonecine, and (**D**) platynecin.

Depending on the structure of the necine base, PAs can be divided into four main types: retronecine, heliotridine, otonecine, and platynecine (Figure 3). Retronecine, heliotridine, and otonecine types include PAs derived from 1,2-unsaturated pyrrolizidine (unsaturated bases), while those of the platynecine type are derived from the saturated base. From a structural point of view, the otonecine type is the most peculiar, as it is derived from a monocyclic base, has a carbonyl group at C-8 and a methylated nitrogen. Retronecine- and heliotridine-type PAs are diastereoisomers at C-7 [5]. Besides these main types of necine bases, there are other types; however, they are of minor significance. 

**Figure 4:**
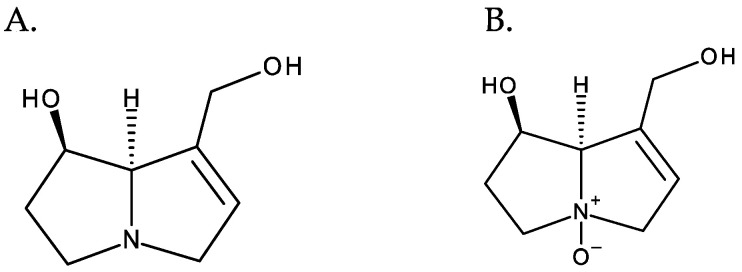
From left to right: (**A**) retronecine and (**B**) retronecine N-oxide.

PAs normally occur in the form of tertiary bases or of their N-oxides, often coexisting in both forms. PANOs are ionic structures resulting from the oxidation of the nitrogen atom of the necine base [10], and they correspond to the most predominant form in plants [1] (Figure 4).

Compared with tertiary bases, which are soluble in non-polar organic solvents and in some polar organic solvents, PANOs are soluble in water, methanol, and other polar organic solvents [5]. These characteristics are relevant in the physiological processes of transport and storage of PAs and have significance for the laboratory procedures of extraction and analysis. All known PAs are capable of forming N-oxide derivatives, except those of the otonecine type [11].

**Figure 5:**
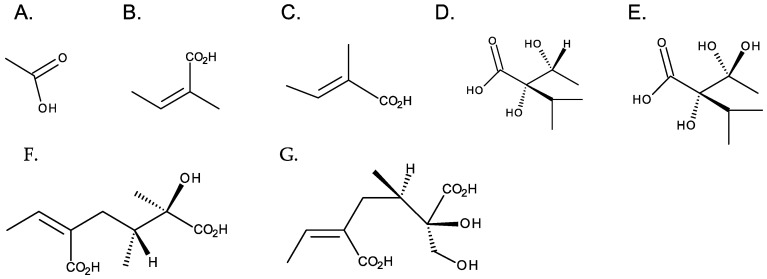
Common necic acids. From left to right: first row—monocarboxylic acids: (**A**) acetic acid, (**B**) angelic acid, (**C**) tiglicic acid, (**D**) trachelantic acid, and (**E**) viridifloric acid; second row: dicarboxylic necic acids: (**F**) senecic acid, and (**G**) isatinecic acid.

Although necine bases are structurally related, necic acids have a wide structural diversity. They are aliphatic, monocarboxylic, or dicarboxylic acids, most with branched carbon chains and hydroxy and alkoxy substituents. Acetic, angelic and tiglic, trachelantic and viridifloric acids (monocarboxylic acids), and senecic and isatinecic acids (dicarboxylic) are examples necic acids (Figure 5). Necic acids with aromatic systems are rarely found [1].

**Figure 6:**
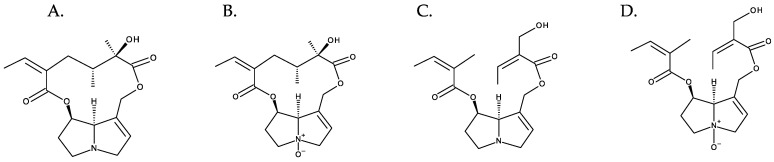
Diversity of PAs exemplified with retronecine derivatives. From left to right: (**A**) senecionine and (**B**) senecionine-N-oxide (cyclic diesters); (**C**) triangularine and (**D**) triangularine-N-oxide (open-chain diesters).

The combination of different necine bases with many necic acids results in the huge diversity of PAs [5]. The N-oxidation of the tertiary nitrogen of the necine base multiplies this diversity [1] (Figure 6). Hundreds of PAs have already been described, and new variants continue to be discovered [1].

It is also common to group PAs into types defined by the binding patterns between necine bases and necic acids [1] or by the plant taxa where they were originally identified. According to Hartmann and Witthe, based on the combination of necine base and the necic acids, it is possible to classify them into five groups. The first and largest group are the senecionine-type PAs, which are found mainly in the *Fabaceae* family and the *Senecionaeae* genus (of the *Asteraceae* family). The triangularine-type PAs represent the second group typical of the *Boraginaceae* family and *Senecioneae* genus. The third type is the lycopsamine-type PAs that can be found in *Boraginaceae*. The fourth group is is monocrolatin-type PAs, predominant in the *Fabaceae* family. Finally, we have the phallenopsin-type PAs that can be found in some species of *Boraginaceae* [1,5,12] (Table 1).

**Figure 7:**
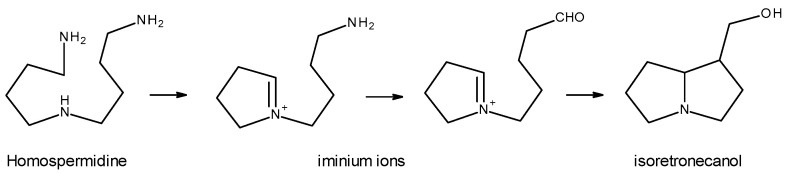
Necine-based biosynthesis (Adapted from: [11]).

The biosynthesis of necine bases begins with the decarboxylation of the amino acids *L*-arginine and *L*-ornithine, which leads to the formation of putrescine [1,5,11]. Next, the formation of the precursor of the necine bases—homospermidine—occurs, for which two theories have been proposed: one that argues that homospermidine results from the condensation of two putrescine molecules [5,11]; another that argues that the formation of homospermidine results from the reaction between a putrescine molecule and another spermidine molecule [1,13]. Regardless of the precursors used, the formation of homospermidine, a key step in the biosynthesis of PAs, is catalyzed by homospermidine synthase. Homospermidine is then cyclized to the corresponding iminium ion, which, in turn, is reduced and cyclized, giving rise to isoretronecanol, trachelantamidine, and rosmarinecine, which are all necine bases. Subsequently, through hydroxylation and dehydration, retronecin and heliotridine are obtained. From retronecin and via subsequent hydroxylations and methylations, otonecine is obtained [11] (Figure 7).

**Figure 8:**
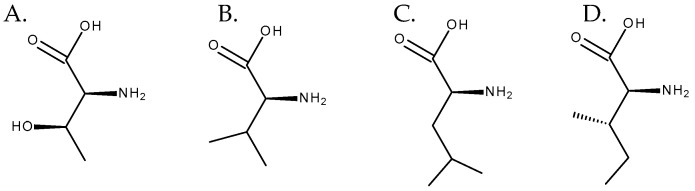
Amino acids involved in the biosynthesis of necic acids. From left to right: (**A**) L-threonine, (**B**) L-valine, (**C**) L-leucine, and (**D**) L-isoleucine.

Although necine bases are synthesized through a common pathway, necic acids can have distinct biosynthetic origins. Some of them, such as acetic acid, are products of the plant’s primary metabolism [1]. The others are mostly derived from aliphatic amino acids, such as *L*-threonine, *L*-valine, *L*-leucine, and *L*-isoleucine [13], the latter having a central role as a precursor of necic acids [14] (Figure 8). Regarding the formation of monocarboxylic acids, such as angelic, tiglic, or sarracinic acids, mainly threonine and isoleucine are involved. The biosynthesis of trachelantic, viridifloric, or senecioic acids mainly involves valine as a precursor. Finally, dicarboxylic acids, such as senecic acid, are mainly formed from isoleucine and threonine [1,5].

The biosynthesis of PAs occurs fundamentally in roots [15]. However, it has also been described that the biosynthetic process can occur in specific young leaves [16]. These compounds are almost exclusively available and stored in the form of N-oxides, which, owing to their high-water solubility, can be easily transported to other plant organs. At any time, PANOs can be reduced to their respective tertiary amines.

It is estimated that approximately 6000 plant species have the capacity to produce one or more of the PAs and PANOs already identified. This fact probably makes PA-producing plants the most common group of toxic plants, capable of affecting both animals and humans. PAs are mostly found in Angiosperms, in the families Asteraceae (tribes Senecioneae—where the genus *Senecio* stands out—and *Eupatorieae*), Boraginaceae (all genera, highlighting the genus *Heliotropium* of the subfamily Heliotropioideae and the genera *Echium* and *Symphytum* of the subfamily Boraginoideae), Fabaceae (genus *Crotalaria*), Orchidaceae, and Apocynaceae [12,14,17].

Some of these plants are used as cover crops for soil improvement, ornamental plants or as animal feed. Among them, there are some species of the genera *Heliotropium* and *Crotalaria*, which are common weeds in crop fields, whereas others are particularly appreciated as melliferous plants (family Boraginaceae) [12].

Most PA-producing plants produce several different PAs that can be found in different concentrations. In a way, the different structural types of PAs are typical of certain taxa, although there are some overlaps. Senecionin-type PAs (such as jacobine, jacoline, jaconine, retrorsine, senecionine and seneciphylline) are characteristic of the tribe Senecioneae (of the Asteraceae family), but also of the Fabaceae family, particularly the genus *Crotalaria*. Lycopsamine-type PAs (which include echimidine, lycopsamine, and vulgarine) appear particularly in the Boraginaceae family, but also in the Eupatorieae tribe of the Asteraceae family. The heliotrine type (which has europine, heliothrine and lasiocarpine as Pas) is typical of the genus *Heliotropium* (from the Boraginaceae family. Monocrotaline-type (for example, 6 ulvene, monocrotaline, and tricodesmine) appear most frequently in the Fabaceae family, more precisely, in the genus *Crotalaria*. The triangularine-type is predominantly found in the Senecioneae tribe and the Boraginaceae family. Phalaenopsin-type PAs are found in the Orchidaceae family. With regard to necic acids, monocarboxylic acids are characteristic of the Boraginaceae family and macrocyclic diesters are characteristic of the Asteraceae family.

**Figure 9:**
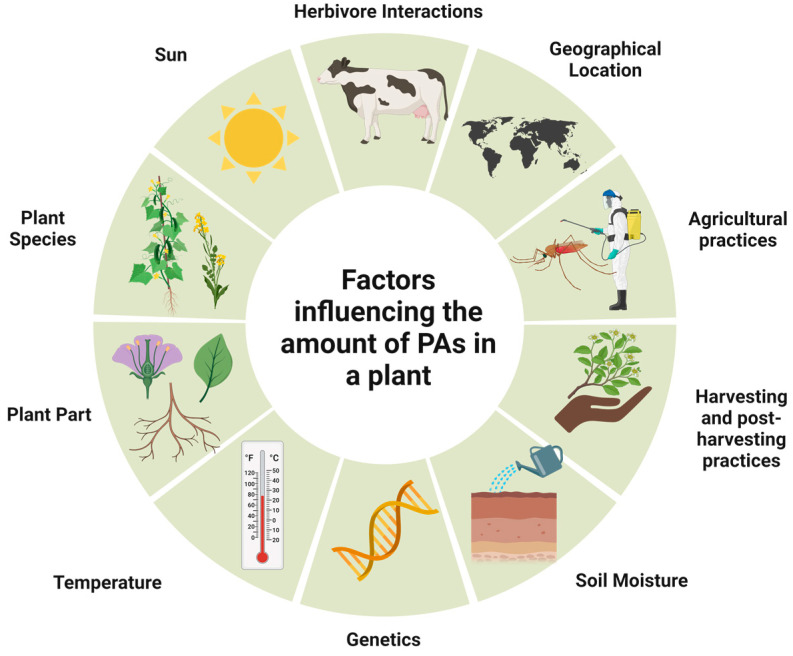
Main factors influencing PAs occurrence and contents in plant material.

The biosynthesis of PAs, which is dependent on genetic variation and heredity, is regulated differently during plant development. The occurrence and number of PAs vary greatly, depending on the species and part of the plant. For example, in the genera *Senecio* and *Eupatorium* (family Asteraceae), PA synthesis is restricted to the roots. It is also influenced by other factors such as climate and soil properties, namely nutrients, water quantity, and herbivore infestations [1] (Figure 9).

### 2.3. Metabolism and Toxicity of PAs

It is estimated that approximately half of the known PAs are toxic [18], meaning that the consumption of plant products originating from numerous species or products derived from them may imply a risk of toxicity. In this regard, the genera *Heliotropium* and *Crotalaria* are particularly relevant, which include the species most commonly associated with poisoning in humans [12].

The toxicological effects of acute exposure of humans and livestock to PAs are known and well documented in the literature. The majority of cases reported in humans are related to the consumption of medicines and herbal dietary supplements, vegetable infusions, cereals, or products derived from them contaminated with seeds from plants producing PAs, honey, and dairy products. These events are mostly related to products produced in regions of Central Asia, Afghanistan, and India, with a direct relationship with periods of meteorological drought, which favor the development of weeds and contamination of crops [12].

Some of the documented cases are related to wheat flour contaminated with *Heliotropium* seeds and qurut—a goat’s milk cheese common in Central Asia—where PAs characteristic of *Heliotropium* and *Crotalaria* species were detected [19].

In Europe, occurrences are rare [17] and the risk of acute poisoning due to PAs is considered low, both by the German Federal Institute for Risk Assessment (BfR) and the European Food Safety Authority (EFSA) [20,21]. However, the potential health risks from chronic exposure to low doses have been the cause of recent concern from European regulatory authorities—EFSA and the European Medicines Agency (EMA) [22], motivated, above all, by the detection of significant amounts of PAs in various foods, as well as in herbal medicines and food supplements [1].

This concern is also because there is little toxicological information on most PAs, and their molecular mechanisms of toxicity are not fully understood [23]. All this apprehension is reinforced by the knowledge, for several decades now, of the acute toxicity, genotoxicity, and carcinogenicity of some of these compounds in laboratory animals [12,21,24,25].

PAs are rapidly absorbed in the gastrointestinal tract, with cutaneous absorption estimated to be of little significance. Compartmental studies have revealed distribution mainly in red blood cells, liver, kidney, lung, and plasma [12]. Because of their lipophilic properties, some PAs are capable of crossing the placental barrier [23].

The catabolism of PAs involves three metabolic pathways.

In general, the metabolization pathways vary according to the necine base; therefore, for retronecine- and heliotridine-type PAs, there are three metabolization pathways:

1. Hydrolysis of the ester groups to form the necine base and the corresponding necic acids.
2. N-oxidation, for conversion to PANOs.
3. Enzymatic hydroxylation of the necine base at the C3 or C8 position to form the respective hydroxynecine derivatives and further C-oxidation, leading to the formation of reactive intermediates pyrolytic esters (DHP) [25,26,27,28].

On the other hand, otonecine-type PAs have only two main metabolic pathways:

1. Hydrolysis of the ester groups to form the corresponding necine bases and necic acids.
2. Formation of the corresponding pyrrolic esters through oxidative N-demethylation of the necine base, which closes the ring by eliminating of formaldehyde and its dehydration [25,28].

The liver is the main organ responsible for metabolism, although small contributions from the lungs and kidneys have been observed. Hydrolysis, which may occur in the transport and distribution process via non-specific blood esterases, occurs mainly through the catalysis of hepatic microsomal carboxylesterases. The products, necine bases and necic acids, are non-toxic and can be immediately conjugated and excreted via urination [11,25].

However, most PAs are oxidized to the corresponding N-oxides through hepatic microsomal monooxygenases: flavin monooxygenase and microsomal cytochrome P450 monooxygenase (CYP450) [11,29]. This N-oxidation is exclusive to the bases of the retronecin and heliotridine types because nitrogen is methylated in the bases of the otonecine type. The N-oxide metabolites are very soluble in water and are therefore easily excreted in urine [20]. However, given that PANOs can be metabolically reconverted into PAs, a cyclical effect may occur [18].

**Figure 10:**
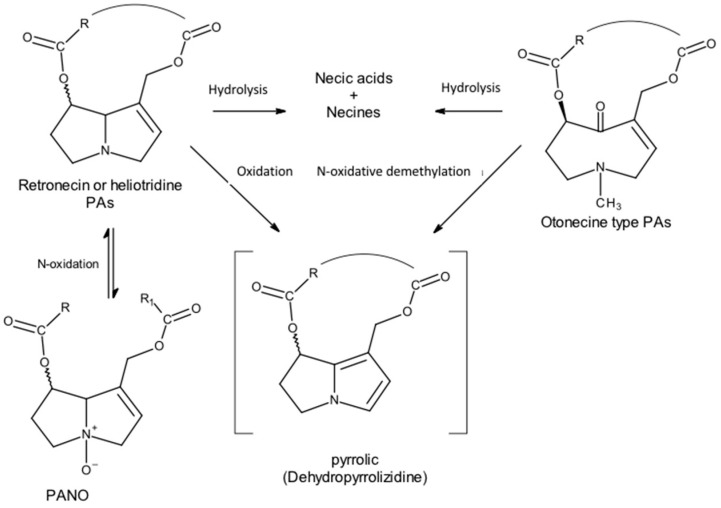
PA metabolism leads to pyrrolic derivatives (Adapted from: [18]).

C-oxidation, particularly of carbon-α, catalyzed by the microsomal monooxygenase CYP450, will convert PAs to reactive toxic pyrrolic metabolites—pyrrolic esters or dehydropyrrolizidine alkaloids (DHPAs) (Figure 10).

PA metabolism is rapid, and metabolites can be detected in the liver and lungs 30–120 min after ingestion [30].

Approximately 80% of ingested PAs are excreted unchanged, predominantly through urine in feces and milk [5].

Not all PAs are toxic, and those that exhibit toxicity have common structural characteristics, among which the double bond between the 1 and 2 positions stands out, being unsaturated PAs (1,2-unsaturated). Therefore, the PAs of the retronecine, heliotridine, and otonecine types stand out as being toxic, and those of the platinecine type as non-toxic. The presence of this double bond is responsible for the level of toxicity in the liver because these compounds need to undergo metabolic activation, to form the highly reactive pyrrole intermediates. The pyrrole intermediates can penetrate the nucleus and react with DNA to form adducts and DNA-protein cross-links that can cause damage, particularly to hepatocytes. Hepatic veno-occlusive disease is caused by the damage that these toxic metabolites cause to the hepatocytes. Briefly, after oral ingestion of PAs, they are rapidly absorbed in the gastrointestinal tract and undergo metabolic activation in the liver [4,5]. Saturated PAs do not undergo metabolic activation in the liver and form water-soluble compounds that are easily excreted [12].

As noted earlier, the liver is the main organ affected by PAs toxicity. HVOD is the main clinical manifestation and is considered a marker of PA toxicity. Some symptoms included vomiting, hepatomegaly, and bloody diarrhea. Poisoning can present itself in three forms: acute, subacute, and chronic, and the point of distinction is the symptoms presented. The acute one is characterized by hemorrhagic necrosis, hepatomegaly, and ascites; in the subacute one, both the ascites and the liver are enlarged, and there is blockage of the hepatic sinuses leading to Sinusiodal Obstruction Syndrome (HSOS); finally, in the chronic one, necrosis, fibrosis and cirrhosis, liver failure, and death are present symptoms [5].

**Figure 11:**
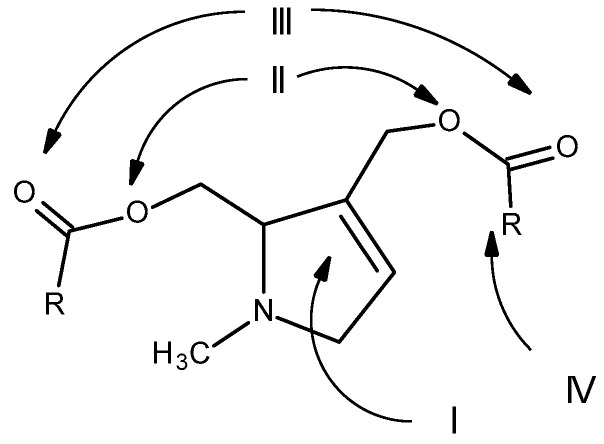
Structural features essential for the toxicity of PAs: (I) double bond at positions 1 and 2; (II) hydroxymethyl substituent at position C1; (III) hydroxyl group at position C7 (III); (IV) presence of a branched necic acid (mono- or dicarboxylic) with at least five carbon atoms (Adapted from: [29]).

Therefore, for PAs to be considered toxic, they must have three minimum structural requirements: the presence of a double bond at positions 1 and 2; a hydroxymethyl substituent at position C1, preferably with a second hydroxyl group at position C7; and the presence of a branched necic acid (mono- or dicarboxylic) with at least five carbon atoms [2] (Figure 11).

## 3. Legal Framework of PAs

In Europe, the European Food Safety Authority (EFSA) is the international body responsible for establishing guidelines to control the occurrence of these compounds in food and feed [31].

The first World Health Organization (WHO) recommendation to reduce exposure to PAs was made in 1988. Later, in 1992, the BfR in Germany issued a document limiting the maximum dose of PAs for internal and external use to 1 µg per day for up to 6 weeks or 0.11 µg per day in the case of prolonged use, for internal use and for external use the limit was 100 µg per day for up to 6 weeks and 10 µg for prolonged use. Subsequently, in 2001, the Australia New Zealand Food Authority determined a provisional intake limit of 1 µg PA/kg/day. This higher intake value, compared with that of BfR, is because, according to the Australian authority, there is no evidence that PAs cause cancer in humans. In 2005, the Dutch National Institute for Public Health set a tolerable intake value of 0.1 μg/kg per day so that no carcinogenic effects were observed. In 2008, the Committee on Toxicity proposed a value of 0.1 µg riddelliine/kg/day for non-carcinogenic PAs, and for PAs considered carcinogenic, the proposed limit is 0.007 µg PA/kg/day for carcinogenic PAs. In 2011, the EFSA, based on the probable genotoxicity and carcinogenicity of PAs and in line with the document published by the BfR in the same year, considered the available information on human intoxication to be insufficient to use as a daily tolerable limit value, setting a maximum limit of 0.007 µg/kg/day for PA intake [2,12,32] (Table 2).

In November 2011, a scientific opinion on the public health risks related to the presence of PAs in food and feed was published by the CONTAM Panel (Scientific Panel on Contaminants in the Food Chain) of the European Food Safety Authority. Subsequently, in 2013, the EFSA submitted a proposal to investigate PA concentrations in food products of animal and plant origin, including herbal infusions and food supplements, in different parts of Europe. After 2 years, the results of the investigation were published. In 2016, the EFSA published a scientific report on the dietary exposure assessment of PAs. In July 2017, the EFSA published a statement on the risks to human health related to the presence of PAs in honey, tea, plant infusions, and food supplements. In this follow-up, the CONTAM Panel established a new reference point of 237 μg/kg body weight per day to assess the carcinogenic risks of pyrrolizidine alkaloids and concluded that there is a possible concern for human health related to exposure to pyrrolizidine alkaloids, mainly for the population who are frequent consumers of tea and herbal infusions, but particularly for younger groups of the population [12,33].

The maximum level of PAs in food used to be regulated by Commission Regulation (EC) No 1881/2006, which is no longer in force. This regulation was updated regarding maximum values for PAs in foodstuffs through Regulation (EC) No. 2020/2040 (no longer in force). However, a new regulation, European Commission Regulation (EU) 2023/915, is in force, and the previous regulation was repealed [33,34,35] (Table 3).

Regulation (EC) No. 2023/915 established the maximum concentration values of PAs in food (Table 3). The maximum concentration value refers to the sum of 21 PAs [35] (Table 3).

## 4. Analytical Methods for Determining PAs

As mentioned earlier, PAs are toxic and can cause harm to the human body. Because of the potential health risks associated with PAs, it is essential to develop robust analytical methodologies for their accurate determination in different types of matrices, including food supplements, herbal teas and infusions, and spices [20]. The growing interest in the determination of PAs in these samples has resulted in the development of new analytical methodologies over the years. However, their high structural diversity and the presence of low concentrations have been challenges for analysts to overcome [4,36,37].

Analytical methods used for the determination of PAs aim to achieve high sensitivity, selectivity, precision, and accuracy, while being cost-effective and validated [38]. The analysis of PAs can be divided into three phases: extraction, separation, and identification, and its success depends on multiple factors [4].

### 4.1. Sampling

Sampling is the first step in determining PAs. This step is crucial to ensure that the sample is representative of the totality of the material to be analyzed [38]. Homogenization is usually the first step after sample collection and can be accomplished by mixing and grinding; depending on the sample, it can be mixed fresh with extraction solvents or frozen and mixed with dry ice and liquid nitrogen. Subsequently, it can be air-dried, vacuum-dried, heated, or freeze-dried [39,40]. There are no documents clarifying the most appropriate method for the determination of PAs. Unlike mycotoxins, which have specific legislation regarding the sampling procedure under Commission Regulation (EC) No 401/2006 of 23 February 2006, toxins from plants, such as PAs, are not regulated [41].

### 4.2. Extraction and Clean-Up Procedures

The extraction step aims to separate the analytes of interest, PAs and PANOs, from the remaining elements of the matrix by reducing and eliminating possible interferences as much as possible before chromatographic analysis [42]. After extraction, a clean-up process is often used [43]. Different extraction techniques have been developed over the past years, and their choice will depend on the type of sample, the alkaloids it is intended to identify, and the complexity of the matrix. PAs are basic polar compounds that are soluble in polar or semipolar organic solvents, such as methanol and acetonitrile, or in a mixture of these solvents with water or acidified aqueous solutions, such as with sulfuric acid [36,37].

The choice of extraction solvent is critical factor in the development of the analytical method and needs to consider the purpose of the study, if it is a phytochemical analysis or risk assessment; for example [37], it is also important to ensure that both, the tertiary forms and the N-oxides are extracted [39].

**Figure 12:**
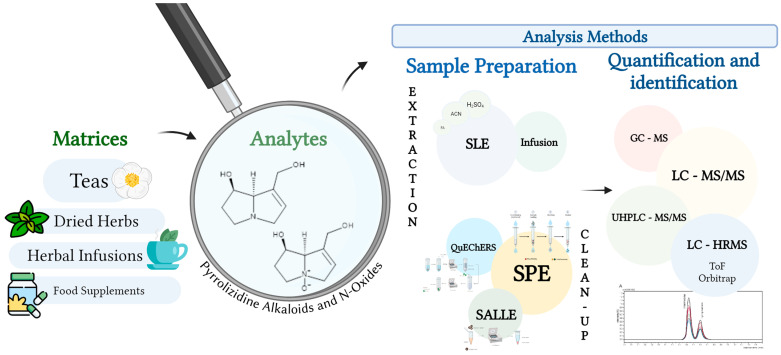
General summary of the extraction, clean-up, quantification, and identification methods used to determine PAs in different matrices.

The most commonly used extraction techniques, (Table 4) are infusion and solid–liquid extraction (SLE) (Figure 12). To increase the efficiency of the extraction techniques, agitation, centrifugation, water bathing, and ultrasonication were applied. Before proceeding to the cleaning process, the samples are centrifuged and filtered to eliminate as many interferences as possible.

After extraction, the matrices generally undergo a clean-up process that purifies the sample and eliminates possible interferents that are still present [40]. Solid-phase extraction (SPE), Quick, Easy, Chip, Effective, Rugged and Safe (QuEChERS), and Salting-out assisted liquid–liquid extraction (SALLE) are the most popular cleaning techniques, according to Table 4 (Figure 12).

SPE is a sample preparation technique that uses a solid sorbent, a cartridge, to selectively retain analytes from a liquid sample matrix. The process involves four phases: conditioning, which aims to activate the solid sorbent using a suitable solvent to allow a suitable phase interface with the sample that will be applied to the SPE cartridge; sample loading, where the sample is passed through the cartridge, one of the most important aspects of this phase is the rate at which the sample passes through the column, which must be slow enough to ensure interaction between the compounds; washing, to remove impurities and ensure that only the PAs and PANOs remain on the adsorbent; and elution, where the PAs/PANOs are eluted from the cartridge with a compatible solvent [44,45]. Han et al. analyzed tea samples using Polymeric Cation Exchange (PCX) cartridges (200 mg/6 mL) conditioned with methanol and deionized water. The analytes were eluted from the SPE column with methanol containing ammonium hydroxide. To optimize the SPE process, the strong cation exchange (SCX) and PCX columns were compared; however, PAs and PANOs were not detected after the clean-up process with the SCX cartridge, so a PCX cartridge was used, which obtained recovery levels between 63.9 and 99.6% for all target compounds [46]. Kwon et al. analyzed the number of PAs in different tea samples, including peppermint, rooibos, chamomile, green tea, and black tea. A mixed-mode cation exchange (MCX) cartridge was used, conditioned with methanol and water, and the samples were then eluted with ammonia in methanol. In this study, both the 500 mg and 150 mg cartridges were studied and showed very similar recovery levels; however, the 150 mg cartridge had a shorter purification time when compared with the 500 mg cartridge; therefore, the 150 mg cartridge was selected for the development of the study [47].

QuEChERS is a sample preparation approach used for the simultaneous detection of several analytes. This method consists of two steps: analyte extraction using partition salts, followed by supernatant cleaning using dispersive solid phase-extraction (dSPE). This method is cost-effective, quick, and simple to perform, has high levels of recovery and sensitivity, and is simple to modify [4,48,49]. In the extraction step, partition salts such as magnesium sulfate (MgSO_4_) and sodium chloride (NaCl) are used to extract the analytes of interest from the sample. In the clean-up step, a primary or secondary amine, (PSA), or octadecyl-bonded silica (C18), adsorbent is used to selectively bind and retain interfering substances, while the target analytes remain in solution [4,44,49,50,51]. León et al. used the QuEChERS method for extraction to determine the presence of PAs and PANOs in tea and herbal infusion samples using MgSO_4_, PSA, C18, and graphitized carbon black (GCB). This procedure led to recoveries between 87 and 111% [52].

Salting-out-assisted liquid–liquid extraction (SALLE) uses the salting-out effect, in which the addition of salts to a solution induces the separation of two immiscible liquid phases. An aqueous salt solution—for example, magnesium sulfate—is added to the sample. The salt will lead to a decrease in the solubility of the PAs and PANOs, and through centrifugation, two phases, the aqueous phase and the organic phase, are separated, in which the analytes are in the organic phase [53,54]. Rizzo et al. analyzed the presence of 118 PAs in food supplements, infusions, honey, and teas. For the analysis of these samples, after pretreatment, the SALLE technique was used for extraction and cleaning. The aqueous extract was salted with magnesium (II) sulfate heptahydrate and sodium sulfate. Recoveries in the range of 61–117% were obtained, with the lowest value in lycopsamine N-oxide. This preparation method showed good repeatability [55].

Table 4 compiles some studies on the presence of PAs and PANOs and the extraction and cleaning techniques used in dietary supplements, herbal teas, and dried herbal teas over the past seven years (2017–2023), with SPE being the most widely used technique [46,47,52,56,57,58,59,60,61,62,63,64,65].

**Table 4:** Extraction procedures to determine PAs/PANOs in dried plants and food supplements and levels of contamination of food matrices.

Type of Sample	Number of Samples	Number of PAs/PANOs	Extraction Conditions	Sample Preparation Procedure	Sampling Period	Recovery (%)	Range of PAs Content Found (µg/kg)	Ref.
Food Supplements	191		Dry Samples: Sonication with 20 mL H_2_SO_4_ 0.05 M Oil Samples: Shaking with 15 mL H_2_SO_4_ 0.05 M	SPE—C18 eluted with 5 mL MeOH; Filtered PCX—SPE eluted with 5 mL 2.5% ammonia in MeOH; Reconstituted with 1 mL MeOH/H_2_O (5/95, *v*/*v*); Filtered	January 2014–April 2015	72–122	<LOD—2410275	[56]
Spices, Tea, Herbals Teas (Tea Infusion and Ice-Tea beverages)	218	30	Dry Samples: 15 mL MeOH + 0.1% FA Infusion Extracts: Infusion with boiling H_2_O;	Dry Samples: SPE with graphitized non-porous carbon; Reconstituted with 1 mL H_2_O/MeOH (80:20). Infusion Extracts and Iced Tea beverages: Basified pH 9–10 with ammonia 28–30%; SPE—C18 eluted with MeOH; Reconstituted with 1 mL H_2_O/MeOH (80:20)	n.d.	70–120	Dry Samples: Range: <LOD—187151 Infusion Samples: Range: <LOD—2106	[63]
Teas and Herbal Teas	18	44	25 mL H_2_SO_4_ in H_2_O	PCX—SPE elute with 10 mL 5% ammoniated MeOH	n.d.	52–152	Range: 0.1–47.9	[66]
Teas Herbal Medicines	5 8	54	Infusion with boiling H_2_O; 20 mL 1% FA in H_2_O	SPE eluted with 6 mL MeOH; Reconstituted with 10% MeOH;	n.d.	73–107	Teas: Range: 30.7–1120 Herbal Medicines: <LOQ—7883	[58]
Teas	50	29	30 mL H_2_SO_4_ 0.005 M; Ultrasonics;	SCX cartridges eluted with 10 mL acetate, MeOH, ACN, ammonia solution and triethylamine (8:1:1:0.3:0.1, *v*/*v*); Reconstituted with MeOH and LiAlH_4_ in THF; Added Dichloromethane and 10% sodium hydroxide; Derivatization;	n.d.	75.1–86.8	Range: 2–6498 Mean: 455	[67]
Spices and Culinary Herbs	305	44	40 mL H_2_SO_4_ 0.05 mM	PCX—SPE eluted with 10 mL 5% ammoniated methanol; Filtered	2014–2017	50–119	Range: 0.1–24,600 Mean: 0.9	[68]
Food Supplements	50	44	Dry samples: 40 mL 0.05 mM H_2_SO_4_ in H_2_O; Liquid Forms: Lyophilized and reconstituted with 40 mL H_2_SO_4_ 0.05 mM in H_2_O Oily capsule content: 20 mL H_2_SO_4_ 0.05 mM in MeOH	SPE	June–July 2018		Solid samples: 0.1–105.1 Liquid Forms: 0.03–2.20	[62]
Peppermint, Chamomile, Nettle and Linden	50	30	10 mL H_2_O followed by 10 mL 0.1% FA in ACN	Ultrasound- Assisted QuEChERS Partition Salts: MgSO_4_, NaCl, TSCDH, DSHCSH 4:1:1:0.5 Clean-UP: Graphene; Filtered	n.d.	61–128	Range: 8–41 Mean: n.d.	[69]
Oregano	23	21	1 mL H_2_O followed by 1 mL ACN	µ-QuEChERS Partition Salts: MgSO_4_, NaCl, TSCDH, DSHCSH 4:1:1:0.5 Clean-Up: dSPE with MgSO_4_ and PSA; Filtered	n.d.	77–96	Range: 334–6375 Mean: 1254	[61]
Herbal Tea and Oregano		33	10 mL MeOH:H_2_O:FA (60:39.6:0.4, *v*/*v*/*v*)	dSPE; Centrifugation	n.d.	Herbal Tea: 80–106 Oregano: 78–117		[59]
Teas, Herbal Teas and Iced Tea Beverages	10	37	Infusion with boiling H_2_O; Extracted with 0.05 M H_2_SO_4_ (3 times)	PCX -SPE eluted with 8.5 mL NH_3_ in MeOH (1.5%, *v*/*v*); Evaporation; Dissolved in MeOH/H_2_O/FA (5:95:0.1%, *v*/*v*, *v*)	2013–2020	70–120	Range: 154–2412 Mean: 422	[64]
Teas (Black, Green, Dark and Chrysanthemum)	385	14	Centrifugation with 10 mL 0.1 M H_2_SO_4_	PCX—SPE eluted with 4 mL MeOH with 0.5% MH_4_OH; Filtered	n.d.	68.6–110.2	Range: <LOQ–151.33 Mean: n.d.	[46]
Teas	290	21	Shaking with 40 mL 0.05 M H_2_SO_4_ in 50% MeOH solution	MCX—SPE eluted with 4 mL H_2_O and 4 mL 2.5% NH_3_ in MeOH; Dissolved with 1 mL MeOH; Filtered	March–September 2017	86.72–101.44	Range: 2–1880 Mean: 230	[47]
Herbal Infusions (Mallow, Calendula and Hibiscus)	9	21	Infusion with 200 mL boiling H_2_O; Filtered	μSPEed	n.d.	79–97	Infusion Samples: Range: 23–113 µg/L Mean: n.d. Dry Samples: Range: 920–4520 µg/L Mean: n.d.	[57]
Tea and Herbs Infusions	11	28	30 mL ACN:H_2_O (75:25, *v*/*v*) with 0.5% FA	QuEChERs Partition Salts: MgSO_4_, CH_3_COONa Clean-UP: dSPE with MgSO_4_, PSA, C18, GCB; Reconstitution with H_2_O:MeOH (95:5, *v*/*v*) with 0.1% FA; Filtered	n.d	87–111	Range: 0.2–2.6 Mean: n.d.	[52]
Herbal Beverage	20	7	5 mL ACN	QuEChERs Partition Salts: NaCl Clean-UP: SPE with PSA; Dissolved with 0.5 mL ACN/H_2_O (5/95, *v*/*v*)	n.d	60.6–120.1	n.d	[59]
Aromatic Herbs (Rosemary, Basil, Thyme and Herbs de Provence)	17	21	1 mL H_2_O followed by 1 mL ACN: Re-Extracted with 0.5 mL ACN before Clean-Up	µ-QuEChERs Partition Salts: MgSO_4_, NaCl, TSCDH, DSHCSH 4:1:1:0.5; Clean-Up: MgSO_4_ and LP-MS-NH_2_; Filtered	n.d	73–105	Range: 49–553 Mean: 262	[70]
Borage	6	22	Sonication with H_2_SO_4_ in aqueous solution; Ultrasonication Bath; Centrifugation	SPE; Dry with N; Dissolved with MeOH:H_2_O (5/95, *v*/*v*); Filtration	n.d	85–121	Range: 87–8165 Mean: n.d.	[65]
Herbal Infusions	60 (Mixed Plants) 25 (Rooibos, Anise, Lemon Balm, Thyme, Peppermint, Lemon Verbena and Mixtures)	28	Infusion with H_2_O; Filtration	SALLE; Dissolved in 200 µL of H_2_O/MeOH 7:3 *v*/*v*	2019–2021	63–117	8.4.1. Range: 865–218,382 Mean: 14,025 8.4.2. Range: 6.5–97.7 Mean: 44	[55]
Teas	51 (*Camellia sinensis* and flavoured teas)	28	Infusion with H_2_O; Filtration	SALLE; Dissolved in 200 µL of H_2_O/MeOH 7:3 *v*/*v*	2019–2021	63–117	Range: 6.9–415.7 Mean: n.d.	[55]
Food Supplements	73 (Plant—based)—41 as solid forms; 8 as syrups/liquid forms	28	Solid Forms: SLE With 0.05 M H_2_SO_4_; Centrifugated Syrups and Liquid Forms: Dilution with H_2_O	Solid Forms: SALLE; Dissolved in 250 µL of H_2_O/MeOH 7:3 *v*/*v*.	2019–2021	63–117	_	[55]
Teas and Herbal Infusion Dietary Supplements	152 52	28	Infusion with 150 mL of boiling H_2_O; Filtered Solid Forms: 10 mL H_2_SO_4_ 0.05 M followed by sonication; Syrups and Liquid Forms: Dilution with H_2_O	SALLE with 1 M of MgSO_4_·7H_2_O, 1.5 M Na_2_SO_4_ and pH 9.6; Reextracted with ACN; Redissolved with H_2_O/MeOH (7:3, *v*/*v*)	n.d.	69–113	-	[71]

Abbreviations: ACN: Acetonitrile; C18: octadecyl bonded silica; DSHCSH: disodium hydrogen citrate sesquihydrate; dSPE: dispersive solid-phase extraction; FA: Formic acid; GCB: graphitized carbon black; ME: methanol; MCX: mixed-mode cation exchanges; n.d.: not described; PCX: polymeric cation exchange; PSA: primary secondary amine; QuEChERS: Quick, Easy, Cheap, Effective, Rugged, Safe; SALLE: salting-out liquid–liquid extraction; SCX: strong cation exchange; SPE: solid-phase extraction; THF: tetrahydrofuran; TSCDH: trisodium citrate dihydrate.

### 4.3. Analytical Methods

The analysis of PAs and their N-oxides is complex due to the wide range of chemical structures of these compounds; therefore, analytical methods of high sensitivity are required to achieve low limits of detection (LOD) and low limits of quantification (LOQ) suitable to monitor the food matrices regarding their compliance with legal limits [12,36].

In November 2020, the European Pharmacopoeia Commission integrated a new chapter (2.8.26 Contaminant pyrrolizidine alkaloids) into the European Pharmacopoeia (Ph.) for the first time, related to contamination with pyrrolizidine alkaloids due to the presence of these analytes and their variation in the composition of herbal medicine matrices and the limits applied. This chapter describes 28 target PAs and allows the use of chromatography procedures coupled to MS/MS or HRMS that meet the validation requirements described in the chapter. The insertion of this chapter in Ph. provided analysts with verification requirements that they could follow when it was necessary to validate the analytical method [72].

However, due to the limitations of LC—MS/MS and the requirement for highly selective and sensitive methods, the United States Pharmacopoeia mentioned that it would be working on a new chapter entitled “Analysis of Contaminant Pyrrolizidine Alkaloids” [73].

The most widely used method is chromatography coupled to mass spectrometry (MS), with liquid chromatography (LC) methods being mostly used because they allow greater sensitivity and can be analyzed directly, while in gas chromatography (GC), if the compounds are not volatile, they require a prior derivatization step. GC has been used in the determination of PAs and PANOs over the years; however, PANOs cannot be analyzed directly, requiring an extra step before chromatographic analysis. This derivatization process is necessary because of their non-volatility, PANOs need to be reduced to their free forms, the PAs. LC offers the advantage of simultaneous detection of PAs and PANOs [8,12,38].

According to Table 5, High-Performance Liquid Chromatography (HPLC) and Ultra High-Performance Liquid Chromatography (UHPLC) are the most-used techniques in all reported studies and the column chosen is C18, varying in its dimensions. The major difference between the HPLC and UHPLC previously mentioned techniques is the particle size of the analytical column and the applied pressure. UHPLC shows higher sensitivity and specificity because it uses analytical column with a stationary phase with a particle size lower than 2 µL, since a smaller size shows significant improvements in separation, resolution, speed, and sensitivity. The dimensions of the column used can also affect efficiency; longer lengths tend to be more efficient. Different mobile phases consisted of mixtures of acidified solutions are generally used. Formic acid (FA) and buffered formate/acetate/ammonium carbonate in organic solvents such as methanol are widely used [40]. Izcara et al. developed a UHPLC—MS/S method to quantify PAs and PANOs present in aromatic plants. A C18 column (100 mm × 2.1 mm, 1.6 µm particle size) was used for this analysis at 25 °C with a flow rate of 0.25 mL/min. The mobile phase (MP) consisted of water and formic acid (A) and methanol with ammonium acetate. LOD and LOQ ranged from 0.1 to 7.5 µg/mL and 0.5 to 25 µg/mL, respectively. The values were validated when in the range of 70–120% [70].

One of the biggest difficulties in the use of liquid chromatography techniques over the past few years in the determination of PAs and PANOs is the co-elution of compounds that occurs when the analytes have equal molecular weight and similar molecular patterns, which hinders the chromatographic separation and respective identification by mass spectrometry. As this is a known problem, the European Commission in Regulation 2023/15 recommends the monitoring of 21 PAs/PANOs and suggests the monitoring of 14 more analytes due to the probability of co-elution with one or more other compound(s). Another problem is that the quantification of these analytes requires the use of reference standards, which makes the analysis only possible for specific compounds and unable to analyze the presence of unknown compounds present [4,8,36].

With regard to compound identification, mass spectrometers play a crucial role in the determination and analysis of PAs and PANOs. Mass spectrometry is a powerful analytical technique that provides information on the molecular weight and structural characteristics of compounds. Molecules are separated according to the ratio between mass and electrical charge (m/z), and the higher the resolving power of the spectrometer, the better its ability to separate two peaks that correspond to similar mass values [74]. It is the most widely used method because of its high selectivity, specificity, and sensitivity. In contrast, ultraviolet (UV) spectrometry is not widely used because of the low specificity of the spectrum for PAs and PANOs, which do not present a characteristic spectrum. Only at 214 nm is it possible to observe a non-specific maximum, which means that their absorption at this wavelength is not unique or distinctive enough to allow the identification of these compounds [12].

Mass spectrometry used in the quantification and detection of PAs and PANOs is usually combined with chromatographic techniques, such as LC or GC, to improve the separation and detection of PAs and PANOs [13]. Kowalczyk et al. developed a GC-MS analysis method that compared the results with those obtained by LC-MS. For this, method a DB-5 MS column (30 m × 0.25 mm, 0.25 µm film thicknesses) was used, and a LOQ value of 1.1 µg/kg and an LOD value of 0.3 µg/kg were obtained. When comparing the results obtained from GC with those obtained from LC, an increase ranging from 10% to 81% in the PAs content was observed [67].

Because PAs and PANOs are polar compounds, electrospray ionization mass spectrometry (ESI-MS) is widely used. The analytes are ionized in solution, making them suitable for chromatographic analysis. The analytes can be negatively or positively charged, in most studies they are in the positive mode. Atmospheric pressure chemical ionization mass spectrometry (APCI-MS) is not widely used for these analytes; it is only used in specific situations where there are PAs and PANOs that may not be efficiently ionized by electrospray ionization (ESI) [38,75].

The choice of analyzer type depends on the purpose of the study, and each analyzer has advantages and disadvantages. The main advantages of LC-MS/MS methods are low limits of detection (LOD) of approximately 1 µg/kg or less and the ability to analyze PAs and PAs and PANOs simultaneously in a single elution [4,12,44].

Triple-Quadrupole (QqQ) is an MS analyzer that has been widely used for the analysis of PAs and PANOs in the studies carried out over the last few years. It has high sensitivity and selectivity and is able to detect trace amounts present in the matrices due also to its ability to perform multiple reaction monitoring (MRM). In conjunction with QqQ, Ion Trap has also been used in the analysis of PAs and PANOs. Similar to QqQ, Ion Trap is also capable of detecting low levels of PAs and PANOs [75].

High-resolution mass spectroscopy (HRMS) has been used more recently and allows a more thorough identification by being able to distinguish compounds of equal molecular weight but different elemental composition. Time-of-Flight (ToF) and Orbitrap are HRMS. ToF-MS is acknowledged for its excellent mass resolution, accuracy, and sensitivity, making it ideal for the characterization and quantification of complex molecules like PAs and PANOs. TOF’s high resolution allows for precise measurement of molecular masses and fragmentation patterns, allowing for the differentiation of various isomers [76]. Orbitrap, similar to TOF, is a high-resolution mass spectrometry technique noted for its high mass accuracy, resolving power, and sensitivity, which allows for the precise measurement of molecular compounds at low concentrations [52].

## 5. PAs Contamination in Food Supplements and Dried Plants

Through observation of Table 6, it is possible to see that the upper range of concentration in PAs in the different matrices, teas, infusions, and food supplements, is well above the legislated range. Mulder et al., in a study conducted to determine the occurrence of PAs in plant-derived foods, determined their concentration in different types of food supplements. It was concluded at the end of the study that 60% of the samples contained significant levels of contamination capable of being detected, and the maximum concentration was detected when analyzing food supplements consisting of PA-producing plants; 2,410,275 µg/kg in dry products [56]. Kwon et al. conducted a study to determine the presence of alkaloids in different teas and infusions, where the maximum concentration was found in the infusion of lemon balm, with a value of 1.88 mg/kg, the maximum allowed value being 400 µg/kg, presenting a value 4.7 times higher than the maximum permitted level at EU [36,47]. Casado et al. analyzed different plants commonly used as infusions, including mallow, calendula, and hibiscus. The maximum concentration was found in the sample belonging to calendula, which was expected, because it belongs to the Astereaceae family and is a PA-producing plant. However, it was not expected to have such measurable values in the mallow and hibiscus samples because they are non-PA-producing plants and belong to the Malvaceae family. These results demonstrate how cross-contamination is present and how high levels of PAs can be found in unexpected food matrices [57].

Food supplements and dried plants are widely consumed by the population. The RASFF is a system set up by the EC to facilitate the rapid exchange of information on food and feed safety issues between national authorities. The presence of PAs in foodstuffs is a reason for authorities to issue an alert notification on the RASFF portal. When an RASFF member (EU Member-States; European Economic Area (EEE) and European Free Trade Association (EFTA) Secretariat; EFSA; EC; Switzerland) detects the existence of a serious risk to public health in a food, feed, or human matrix, the EC receives this notification, verifies it, and issues an alert to the other members. When countries receive the alert issued, the members check whether it is relevant to them or not. If there is a product on the market, they are able to detect it and, on the basis of the information contained in the notification, take the necessary measures, which are then transmitted to the other members. Some measures that can be taken include withdrawal or recall of the product, informing the public, and re-dispatching to the place of origin [77].

In the RASFF Portal, since 2020, 77 notifications have been issued regarding the contamination of foodstuffs with PAs. Of the 77 notifications, only two were considered non-serious, eight were undecided, and the remaining 67 were considered serious risks. The main notified matrices were dried oregano and cumin. Turkey is the country with the highest origin of notifications.

The sample with the highest levels of PAs originated from Italy and was a sample of borage, where a concentration of over 59,999 μg/kg was detected. This value far exceeds the maximum EU legislated value for this food matrix (1000 μg/kg). Despite this matrix having the highest content of PAs, there is only one notification for borage. Dried oregano and cumin predominate the notifications. For oregano, the highest PA content found was in a sample originating from Greece and resulted in 30,313 μg/kg, the maximum permitted limit being 1000 μg/kg. Regarding cumin samples, the sample with the highest PA content was 55,176 µg/kg with a maximum permitted value of 400 µg/kg.

The analyses resulting from notifications on the RASFF portal show that the presence of PAs in these food matrices is much higher than that permitted. The EC has established Implementing Regulation (EU) 2022/913 [78].

Laying down rules on the entry of certain foodstuffs into the EU from third countries. Turkey, the country of origin with the highest number of notifications, is present in the regulation with a control frequency of 10% for both cumin and oregano.

Analyzing Table 6 regarding the notifications of the RASFF portal, we conclude that the legislation is not being complied with and that strict control is urgently needed to ensure food safety to avoid animal and human exposure to PAs in order to avoid future consequences.

## 6. Conclusions: Challenges and Future Perspectives

PAs are considered to be toxic secondary metabolites produced by various plant families. Concern about their toxicity has led to several studies being carried out over the last few years reporting on the toxicity of these compounds in different matrices, such as teas and infusions, food supplements, and dried herbs. The most widely used extraction techniques are infusion and solid–liquid extraction. Once extracted, they are usually subjected to a cleaning process, with the SPE technique being the most widely used. Once the sample is free of impurities, it is subjected to chromatographic analysis, GC-MS, or LC-MS, the latter being the most common and the one described in the pharmacopeia. Despite being the most widely used, this technique has some limitations, such as the co-elution of compounds, which make it difficult to identify them.

Since the contamination of food matrices with these compounds is unpredictable, minimizing the occurrence of these compounds must result from prevention and control. Several challenges need to be overcome, and these are related to various aspects, such as toxicity assessment, analytical methodologies, regulatory measures, and good sustainable practices. Over the years, analytical methods have been challenged to find more sensitive and selective techniques for the detection and quantification of these compounds in complex matrices. However, advances in mass spectrometry seem to be overcoming this difficulty, and the use of high-resolution detectors, such as ToF and Orbitrap, is expected to grow exponentially in the coming years.

Harmonization of legislation seems to be another challenge to overcome. Global harmonization regarding maximum limits for PAs in food matrices is important because contamination levels in various matrices have been exceeded on a large scale. Harmonized regulations can help ensure food safety for consumers and facilitate international trade. In the long term, notifications on the RASFF portal will decrease. Therefore, the implementation of good agricultural practices to standardize a planting method for species to be used later in food should be considered to minimize plant variability, since, as PAs occur naturally in plants, their concentration can vary greatly depending on the stage of growth, the environmental conditions to which they are subjected, and their geographical location.

Finally, consumers need to be made aware of the toxicity of these compounds. Educate the consumer to understand the label to make an informed choice and alert them to the importance of buying products from reputable stores.

## Figures and Tables

**Table 1:** Examples of PA-producing plants and their PAs.

Family	Genus	Species (e.g.)	Type of PA	PAs
Fabaceae	*Crotalaria* spp.	*C. albida* *C. assamica* *C. pallida* *C. sessiliflora*	Monocrotalin-type	Monocrotaline Aucherine
			Senecionine-type	Senecionine Platyphylline Rosmarinine Senecivernine Nemorensine
Asteraceae	*Senecio* spp.	*S. jacobaea* *S. vulgaris* L. *S. nemorensis* L. *S. argunensis* *S. integrifolius* (L.) *S. scandens* *S. longilobus*	Triangularine-type	Triangularine Sarracine Macrophylline
	*Eupatoriae* spp.	*E. cannabinum* *E. chinense* *E. japonicum* *E. fortunei*	Lycopsamine-type	Acetylechimidine and isomers Echimidine and isomers
Boraginaceae	*Heliotropium* spp.	*H. arborescens* *H. indicum*	Lycopsamine-type Phalaenosine-type	Europine Heliotrine Lasiocarpine

**Table 2:** Proposal of different regulatory bodies for exposure limits to PAs.

Regulatory Body	Limits	
BfR (1992)	Internal use: Up to 6 weeks: 1 µg PA/day Prolonged use: 0.11 µg PA/day	External use: Up to 6 weeks: 100 µg PA/day Prolonged use: 10 µg PA/day
	10 µg PA/day if consuming coltsfoot leaves as a tea infusion	
Australia New Zealand Food Authority (2001)	1 µg PA/kg/day	
Dutch National Institute for Public Health and the Environment (2005)	0.1 µg PA/kg/day	
Committee on Toxicity of Chemicals in Food, Consumer Products and Environment (2008)	0.1 µg riddelliine/kg/day for non-carcinogenic PAs 0.007 µg PA/kg/day for carcinogenic Pas	
EFSA (2011)	0.007 µg PA/kg/day	

**Table 3:** Maximum permitted levels of PAs in food products according to EU legislation (Reg. EC No. 2023/915).

Food Product	Maximum Concentration Level (μg/kg) ^1^
Herbal Infusions of rooibos, anise, lemon balm, chamomile, thyme, peppermint, lemon verbena (dried product) and mixtures exclusively composed of dried herbs	400
Other herbal infusions (dried product) excluding those mentioned above	200
Tea (Camellia sinensis) and flavoured tea (Camellia sinensis) (dried product)	150
Tea (Camellia sinensis) and flavoured tea (Camellia sinensis) and herbal infusions for infants and young children (dried product)	75
Tea (Camellia sinensis) and flavoured tea (Camellia sinensis) and herbal infusions for infants and young children (liquid)	1.0
Food supplements containing herbal ingredients including extracts with the exception of the pollen-based food supplements, pollend and pollen products	400
Pollen based food supplements. Pollen and pollen products	500
Borage leaves (fresh, frozen) placed on the market for the final consumer	750
Borage, lovage, marjoram and oregano (dried) and mixtures exclusively composed of these dried herbs	1000
Dried herbs except those mentioned above	400
Cumin seeds (seed spice)	400

^1^ Refer to the maximum total concentration of PAs and PANOs that can be found in the corresponding food.

**Table 5:** Liquid chromatography analytical methodologies to determine pyrrolizidine alkaloids in dried herbs and food supplements.

Analytical Technique	Conditions	Analytical Column	Type of Mass Spectrometer and Ionization	LOD and LOQ (µg/kg)	Ref.
UHPLC—MS/MS	MP: A: 0.1% FA + 5 mM NH_4_HCO_2_ in H_2_O; B: 0.1% FA + 5 mM NH_4_HCO_2_ in MeOH/H_2_O (95:5, *v*/*v*) Flow Rate: 0.3 mL/min Injection Volume: 10 µL	C18 column (100 × 2.1 mm, 1.9 µm) Oven: 40 °C	TQ—MRM ESI (+)	Dry Samples LOD: 0.3–2.3 LOQ: n.d Oil Samples: LOD: 0.9–3.8 LOQ: n.d	[56]
UPLC-MS/MS	MP: A: H_2_O with 0.1% ammonia; B: ACN; Flow Rate: 0.4 mL/min Injection Volume: 10 µL (plant extracts) and 5 µL (infusion extracts)	C18 (100 × 2.1 mm, 1.7 µm particle size) Oven: 45 °C	TQ—MRM ESI (+)	LOD: - LOQ: 0.1–1 (dry samples); 0.01 (infusion extracts);	[63]
LC—MS/MS	M.P: A: H_2_O, MeOH/H_2_O (10/90,5/95, *v*/*v*) or ACN/H_2_O (10/90, 5/95, *v*/*v*): B: MeOH/H_2_O or ACN/H_2_O (95/5 *v*/*v*) or (90/10 *v*/*v*), C: MeOH/H_2_O (90/10, *v*/*v*) or ACN/H_2_O (90/10, *v*/*v*) Flow Rate: 0.4 mL/min Injection Volume: 20 µL	C18 (150 × 2.1 mm, 5 µm particle size) Oven: 30 °C	TQ—MRM ESI (+/−)	LOD: 0.1–7.0 LOQ: 0.1–27.9	[66]
UPLC—MS/MS	MP: A: 10 mM (NH₄) ₂CO₃ pH 9 in H_2_O; B: ACN; Flow Rate: 400 µL/min Injection Volume: 2 µL	C18 column (150 mm × 2.1 mm, 1.7 µm particle size) Oven: 50 °C	TQ—MRM ESI (+)	Teas LOD: 0.01–0.02 LOQ: 0.05 Herbal Medicines: LOD: 1–2 LOQ: 4–5	[58]
GC—MS	Flow Rate: 0.8 mL/min Injection Volume: 2 µL	DB—5 MS (30 × 0.25 mm, 0.25 film thicknesses Oven: 250 °C	MSDI	LOD: 0.3 LOQ: 1.1	[67]
HPLC—MS/MS	MP: A: 0.1%FA + 5 mM NH_4_HCO_2_ in H_2_O; B: 0.1%FA + 5 mM NH_4_HCO_2_ in ACN/H_2_O (95/5, *v*/*v*); Flow Rate: 0.4 mL/min Injection Volume: 20 µL	C18 column (150 mm × 2.1 mm) protected by C18 2.1 mm Oven: 30 °C	TQ	LOD: <0.1–2.6 LOQ: n.d.	[62]
LC—MS/MS	MP: A: 0.5% FA + 2 mM NH_4_HCO_2_ in H_2_O; B: 0.5% FA + 2 mM NH_4_HCO_2_ in MeOH low Rate: 0.3 mL/min Injection Volume: 5 µL	C18 column (100 × 2.1 mm, 1.9 µm) Oven: 40 °C	Qtrap MRM ESI (+)	LOD: n.d. LOQ: 1	[69]
UHPLC-MS/MS	MP: A: 0.2% FA + 5 mM NH₄CH₃CO₂ in H_2_O; B: 10 mM NH₄CH₃CO₂ in MeOH; Flow Rate: 0.25 mL/min Injection Volume: 2 µL	C18 Column (100 mm × 2.1 mm, 1.6 µm particle size). Oven: 25 °C	Ion-Trap ESI (+)	LOD: 0.1–7.5 LOQ: 0.5–25	[61]
RP—UHPLC—MS/MS	MP: 0.2% FA + 2 mM NH_4_HCO_2_ in H_2_O; B: 0.2% FA + 2 mM NH_4_HCO_2_ in MeOH; Flow Rate: 0.25 mL/min Injection Volume: 1 µL MP: A: 0.1% FA + 5 mM NH_4_HCO_2_ in H_2_O; B: 0.1% FA + 5 mM NH_4_HCO_2_ in ACN:H_2_O (95:5, *v*/*v*); Flow Rate: 0.3 mL/min Injection Volume: 1 µL	C18 Column (150 mm × 2.1 mm, 1.6 µm particle size). Oven: 50 °C ACQUITY UPLC BEH Amide (100 mm × 2.1 mm, 1.7 µm particle size) Oven: 40 °C	Qtrap MRM ESI (+)	LOD: LOQ: 0.5–10	[59]
LC—HR—MS	MP: A: 0.1% FA in H_2_O; B: 0.1% FA in MeOH; Flow rate: 0.2 mL/min Injection Volume: 5 µL	C18 (125 × 2 mm, 3.5 µm) Oven: 30 °C	Qq-TOF ESI (+)	LOD: 0.1–5 LOQ: n.d.	[64]
UHPLC-MS/MS	MP: A: 0.1% FA + 1 mM NH_4_HCO_2_ in MeOH; B: 0.1% FA + 1 mM NH_4_HCO_2_ in H_2_O; Flow Rate: 0.25 mL/min Injection Volume: 3 µL	T3 (100 × 2.1 mm, 1.8 µm particle size) Oven: 40 °C	TQ ESI (+) MRM	LOD: 0.001–0.4 LOQ: 1–5	[64]
LC—MS/MS	MP: A: 5 mM NH_4_HCO_2_ + 0.1% FA in H_2_O; B: 5 mM NH_4_HCO_2_ + 0.1% FA in 95% MeOH; Flow Rate: 0.3 mL/min Injection Volume: 10 µL	C18 (100 mm × 2.1 mm, 3.5 µm) Oven: 40 °C	Tandem MS ESI (+) MRM	LOD: 0.1–3.0 LOQ: 0.3–9.0	[47]
UHPLC-MS/MS	MP: A: 10 mM NH₄CH₃CO₂ in MeOH; B: 5 mM NH₄CH₃CO₂ in H_2_O; Flow Rate: 0.25 mL/min Injection Volume: 5 µL	C18 Column (100 mm × 2.1 mm, 1.6 µm particle size). Oven: 25 °C	Ion-Trap ESI (+)	LOD: 0.1–0.3 µg/L LOQ: 0.3–1 µg/L	[57]
LC—HRMS	MP: A: 0.1% FA in H_2_O; B: 0.1% FA in MeOH; Flow rate: 300 µL/min Injection Volume: n.d.	C18 column (100 × 2.1 mm, 1.9 µm) Oven: 40 °C	Q- Orbitrap H-ESI (+) PRM	LOD: n.d. LOQ: 5	[53]
UPLC-IM-QTOF	MP: A: 0.1% FA in H_2_O; B: 0.1% FA in ACN; Flow Rate: 0.45 mL/min Injection Volume: 5 µL	C18 column (2.1 mm × 100 mm; 1.7 µm) Oven: 50 °C	QTOF ESI (+)	LOD: n.d. LOQ: 1–20	[59]
UHPLC-MS/MS	MP: A: 0.2% FA + 5 mM NH_4_HCO_2_ in H_2_O; B: NH_4_CO_2_ in MeOH; Flow Rate: 0.25 mL/min Injection Volume: 2 µL	C18 Column (100 mm × 2.1 mm, 1.6 µm particle size). Oven: 25 °C	Ion-Trap ESI (+) MRM	LOD: 0.4–3.0 LOQ: 1.2–10	[69]
LC—MS/MS	MP: A: 0.1% FA + 5 mM NH_4_HCO_2_; B: ACN Flow Rate: 0.3 mL/min Injection Volume: 2 uL	C18 Column (150 mm × 2.1 mm, 1.6 µm particle size) with a pre-column Fully Porous Polar C18 (2.1 mm ID columns) Oven: 25 °C	DAD coupled a Triple TOF ESI (+)	LOD: n.d. LOQ: 25–50	[65]
UHPLC—HRMS/MS	MP: A: 0.1% HCOOH in H_2_O; B: 0.1% HCOOH in ACN; Flow rate: 400 µL/min	C18 (2.1 × 100 mm, 1.6 μm) a Oven: 40 °C	Q-Exactive HESI-II (+)	Solid Matrices: LOQ: 0.1–2.1 LOD: 0.0 Infusions and Teas: LOQ: 1–12 LOD: 0.0	[55]
UHPLC—HRMS/MS	MP: A: 0.1% FA in H_2_O; B: 0.1% FA in ACN; Flow rate: 400 µL/min Injection Volume: 5 µL	C18 (2.1 × 100 mm, 1.6 μm) a Oven: 40 °C	Q-Exactive HESI-II (+)	LOD: 0.6–30 LOQ: n.d.	[71]

Abbreviations: ACN: Acetonitrile; C18: octadecyl bonded silica; DAD: diode array detection; ESI: electrospray ionization; GC: gas chromatography; HPLC: high performance liquid chromatography; HRMS: high-resolution mass spectrometry; IT: ion-trap; LOD: limit of detection; LOQ: limit of quantification; MP: mobile phase; MeOH: methanol; MRM: multiple reaction monitoring; MS: mass spectrometry; MS/MS: tandem mass spectrometry; QToF: quadrupole time-of-flight; Qtrap: hybrid triple quadrupole-linear ion trap; RP: reverse phase; TQ: triple quadrupole; UHPLC: Ultra-High Performance Liquid Chromatography.

**Table 6:** RASFF notifications due to PA contamination from 2020 to 2023.

Date (Day/Month/Year)	Country	Origin Country	Product	Levels of Contamination (μg/kg)
11/07/2023	Poland	India, Poland	Ground Cumin	1217
26/06/2023	Belgium	Turkey	Ground Cumin	23,813
26/06/2023	Greece	Turkey	Ground Cumin	8281
21/06/2023	Germany	Turkey	Cumin	13,600
14/06/2023	Belgium	Turkey	Cumin	2259 ± 890
14/06/2023	Luxembourg	Spain	Cumin seeds	717 ± 108
12/05/2023	Poland	Poland	Herbata Loyd Earl grey	240 ± 40
02/05/2023	Bulgaria	Turkey	Ground Cumin	1553.4
12/04/2023	Sweden	Turkey	Dried Oregano	2263
28/03/2023	Germany	Germany and Greece	Organic oregano	24,000
28/03/2023	Czech Republic	Poland	Dried Oregano	1448
22/03/2023	Germany	Greece	Oregano	17,000
07/03/2023	France	Belgium and France	Cumin seeds	10,000
17/02/2023	Ireland	India	Cumin whole	521.1 ± 87.9
17/02/2023	Ireland	n.d.	Dried Oregano	n.d.
07/02/2023	Germany	Italy	Borage	>59,999
03/02/2023	Belgium	Belgium	Ground Cumin	16,596
30/01/2023	Belgium	France	Ginkgo Biloba extract	702
27/01/2023	Belgium	n.d.	Camomile tea	2470
27/01/2023	France	Turkey	Cumin seeds	1148.9 ± 574.4
				660.9 ± 330.5
				563.7 ± 281.9
23/01/2023	Netherlands	France	Licorice root grinded	1558
12/01/2023	Norway	Moroco	Hayatea herbal tea with peppermint, mentha pulegium (pennyroyal), sage, verveine, and oregano	11,608.3
10/01/2023	Romania	Poland	Black Tea	700
04/01/2023	Italy	Turkey	Dried Oregano	n.d.
03/01/2023	Poland	Poland	Pollen	1187 ± 301
29/12/2022	Spain	Turkey	Cumin	7290 ± 3650
16/12/2022	Belgium	n.d.	Ground cumin	5298
				2926
13/12/2022	Poland	Turkey	Dried Oregano	13,921 ± 2735
13/12/2022	Greece	n.d.	Cumin	17,512
01/12/2022	Germany	India	Ground Cumin	4040 ± 1620
22/11/2022	Belgium	Afganistan and France	Ground Cumin	23,899
				14,249
21/11/2022	Belgium	Turkey	Oregano	1983.5
17/11/2022	France	Turkey	Dried Oregano	5174 ± 2587
15/11/2022	Poland	Turkey	Dried Oregano	8236 ± 1564
03/11/2022	Belgium	n.d.	Ground Cumin	3697 ± 1395
				10,118 ± 3915
02/11/2022	Netherlands	Greece	Oregano	30,313
19/10/2022	Italy	Turkey	Dried Oregano	5591 ± 1177
10/10/2022	Switzerland	Turkey	Ground Cumin	4436
10/10/2022	Bulgaria	Turkey	Dried Oregano	>2500
25/08/2022	Ireland	Turkey	Ground Cumin	1191.4 ± 197.8
10/06/2022	Sweden	Turkey	Cumin	12,350
				10,560
12/05/2022	Bulgaria	Turkey	Ground Cumin	>2500
10/05/2022	Bulgaria	Turkey	Dried Oregano	2154
07/05/2022	Bulgaria	Turkey	Dried Oregano	2644.1
24/04/2022	Bulgaria	Turkey	Cumin	1505.4
22/04/2022	Ireland	Turkey	Cumin	1723.8
				4810.6 ± 801.4
30/03/2022	Finland	Turkey	Dried Oregano	6970
07/03/2022	Spain	Turkey	Cumin seeds	50,000
01/03/2022	Czech Republic	Turkey	Ground Cumin	11,907.7
05/01/2022	Netherlands	Spain	Pollen	880
23/12/2021	Denmark	Spain	Oregano	14,000 ± 5000
22/12/2021	Denmark	Uzbekistan	Camomile tea	5400
28/10/2021	Germany	Turkey	Oregano	2785
				2568
19/10/2021	Germany	Turkey	Cumin seeds	9474
02/06/2021	Switzerland	Turkey	Oregano	4879
20/05/2021	Germany	Turkey	Oregano	2079
14/05/2021	Germany	Turkey	Organic cumin	10,483.39
07/05/2021	Germany	Turkey	Cumin	10,906.77
05/05/2021	Germany	Turkey	Cumin	10,406.94
01/04/2021	Germany	Czech Republic	Herbal Tea	2928.1
26/03/2021	Switzerland	Turkey	Oregano	8895
12/02/2021	Germany	Turkey	Ground Cumin	27,500 ± 970
21/01/2021	Germany	Netherlands	Ground Cumin	21,200 ± 5300
24/12/2020	Switzerland	Turkey	Ground Cumin	9948
23/12/2020	Switzerland	Turkey	Ground Cumin	5786
				20,377
23/12/2020	Switzerland	Turkey	Ground Cumin	5522
04/12/2020	Germany	Turkey	Cumin	11,700 ± 2900
18/11/2020	Germany	Netherlands	Ground cumin	55,176
18/11/2020	Germany	Lebanon	Cumin	22,000
				18,900
20/08/2020	Germany	Egypt	Anise seeds	12,184
				15,114
				1206 ± 188
30/06/2020	Switzerland	Turkey	Organic cumin	29,120
30/04/2020	Germany	Turkey	Organic cumin	56,100
24/04/2020	Denmark	Turkey	Ground Cumin and Dried Oregano	15,000
				7200
30/03/2020	Germany	Turkey	Oregano	6620
11/02/2020	Belgium	Poland	Camomile tea	530
05/02/2020	Germany	Turkey	Oregano	16,962 ± 8481
04/02/2020	Germany	Turkey	Rubbed oregano	8836

## Data Availability

Data sharing is not applicable to this paper.

## Abbreviations

ACN	Acetonitrile
APCI-MS	Atmospheric-pressure chemical ionization mass spectrometry
BfR	German Federal Institute for Risk Assessment
C18	octadecyl bonded silica
CONTAM	Scientific Panel on Contaminants in the Food Chain
CYP450	cytochrome P450 monooxygenase
DAD	diode array detection
DHP	reactive intermediates pyrolytic esters
DHPAs	dehydropyrrolizidine alkaloids
DNA	Deoxyribonucleic acid
DSHCSH	disodium hydrogen citrate sesquihydrate
dSPE	dispersive solid phase ex-traction
EC	European Commission
EEE	European Economic Area
EFSA	European Food Safety Authority
EFTA	European Free Trade Association
EMA	European Medicines Agency
ESI	electrospray ionization
EU	European Union
FA	Formic Acid
GC	Gas Chromatography
GCB	graphitized carbon black
HPLC	High performance liquid chromatography
HRMS	High-Resolution Mass Spectrometers
HSOS	Sinusiodal Obstruction Syndrome
HVOD	Hepatic Veno-Occlusive Disease
IARC	International Agency for Research on Cancer
LC	Liquid Chromatography
LOD	Limit of Detection
LOQ	Limit of Quantification
MCX	Mixed-mode Cation Exchange
MeOH	Methanol
MP	Mobile Phase
MRM	Multiple Reaction Monitoring
MS	Mass Spectrometry
PA	Pyrrolizidine Alkaloid
PANOs	Pyrrolizidine Alkaloid N-oxide
PCX	Polymeric Cation Exchange
PSA	Primary Secondary Amine
QqQ	Triple Quadrupole
QuEChERS	Quick, Easy, Chip, Effective, Rugged and Safe
RASFF	Rapid Alert System for Food and Feed
RP	Reverse Phase
SALLE	Salting-out Assisted Liquid–Liquid Extraction
SCX	Strong Cation Exchange
SLE	Solid-Liquid Extraction
SPE	Solid-Phase Extraction
THF	Tetrahydrofuran
ToF	Time-of-Flight
TQ	Triple Quadrupole
TSCDH	Trisodium Citrate Dihydrate
UHPLC	Ultra-high-performance liquid chromatography
UPLC	Ultra-Performance Liquid Chromatography
UV	Ultraviolet
WHO	World Health Organization
